# Research Progress on the Degradation of Organic Pollutants in Water by Activated Persulfate Using Biochar-Loaded Nano Zero-Valent Iron

**DOI:** 10.3390/molecules29051130

**Published:** 2024-03-03

**Authors:** Hai Lu, Xiaoyan Wang, Qiao Cong, Xinglin Chen, Qingpo Li, Xueqi Li, Shuang Zhong, Huan Deng, Bojiao Yan

**Affiliations:** 1 Key Laboratory of Songliao Aquatic Environment, Ministry of Education, Jilin Jianzhu University, Changchun 130118, China; luhai@jlju.edu.cn (H.L.); wangxiaoyan@student.jlju.edu.cn (X.W.); congqiao@jlju.edu.cn (Q.C.); chenxinglin@student.jlju.edu.cn (X.C.); liqingpo@student.jlju.edu.cn (Q.L.); 2 Urban Construction College, Changchun University of Architecture, Changchun 130607, China; lixueqi@student.jlju.edu.cn; 3 Key Laboratory of Groundwater Resources and Environment, Ministry of Education, Jilin University, Changchun 130021, China; zhongshuang@jlu.edu.cn; 4 College of Visual Arts, Changchun Sci-Tech University, Changchun 130600, China; 100343@cstu.edu.cn

**Keywords:** biochar-loaded nano zero-valent iron, persulfate, organic pollutants, influencing factors, reaction mechanism, research progress

## Abstract

Biochar (BC) is a new type of carbon material with a high specific surface area, porous structure, and good adsorption capacity, which can effectively adsorb and enrich organic pollutants. Meanwhile, nano zero-valent iron (nZVI) has excellent catalytic activity and can rapidly degrade organic pollutants through reduction and oxidation reactions. The combined utilization of BC and nZVI can not only give full play to their advantages in the adsorption and catalytic degradation of organic pollutants, but also help to reduce the agglomeration of nZVI, thus improving its efficiency in water treatment and providing strong technical support for water resources protection and environmental quality improvement. This article provides a detailed introduction to the preparation method and characterization technology, reaction mechanism, influencing factors, and specific applications of BC and nZVI, and elaborates on the research progress of BC-nZVI in activating persulfate (PS) to degrade organic pollutants in water. It has been proven experimentally that BC-nZVI can effectively remove phenols, dyes, pesticides, and other organic pollutants. Meanwhile, in response to the existing problems in current research, this article proposes future research directions and challenges, and summarizes the application prospects and development trends of BC-nZVI in water treatment. In summary, BC-nZVI-activated PS is an efficient technology for degrading organic pollutants in water, providing an effective solution for protecting water resources and improving environmental quality, and has significant application value.

## 1. Introduction

Organic pollutants in water mainly come from industrial wastewater, agricultural discharge, domestic sewage, and atmospheric sedimentation [1]. These pollutants include volatile organic compounds, halogenated hydrocarbons, pesticides, dyes, surfactants, drugs, and personal care products [2]. They pose a severe threat to the water environment and human health, affecting the growth and reproduction of aquatic organisms, reducing water quality and the utilization value of water resources, increasing the difficulty and cost of water treatment, and even causing diseases such as cancer [3,4].

With the development of industry and agriculture, there are more and more organic pollutants in water [5]. These organic pollutants can be divided into two categories, natural and synthetic, and mainly come from industrial wastewater, agricultural discharge, domestic sewage, and atmospheric deposition [6]. These pollutants are various, including volatile organic compounds, halogenated hydrocarbons, pesticides, dyes, surfactants, drugs, and personal care products [7]. Most organic substances in water are prone to bioaccumulation, which affects the growth and reproduction of aquatic organisms [8]. Some of them have low concentrations and are not easy to degrade, and some of them have teratogenic, carcinogenic, and mutagenic toxic effects, which reduce the utilization value of water quality and water resources, and even cause great potential harm to human beings and the environment [9]. In addition, if the concentration of organic pollutants in the water source exceeds the standard, the by-products brought by chloride disinfection in the water will increase, and the usage and residual amount of coagulant in the water will rise, which will increase the difficulty and cost of water treatment [10]. In recent years, with the continuous enhancement of people’s awareness of environmental protection and the continuous improvement of analysis and detection technology, the present situation of organic pollution to the environment has aroused widespread concern [11]. Therefore, it is very important to develop green and efficient water treatment technology to reduce the serious threat of organic pollutants to the water environment and human health [6].

For a water source polluted by organic substances, especially toxic and harmful substances dissolved in water, conventional treatment processes (coagulation, sedimentation, filtration, and disinfection) cannot meet the purification requirements [12]. Therefore, pretreatment and advanced treatment are added to conventional treatment. The main targets of pretreatment and advanced treatment are organic pollutants in water, which are mainly used in drinking water treatment plants [13]. Pretreatment methods mainly include powdered activated carbon adsorption, ozone or potassium permanganate oxidation, biological oxidation, and so on [14]. Advanced treatment mainly includes the following methods: granular activated carbon adsorption method, ozone-granular activated carbon combined method, or biological activated carbon method, chemical oxidation method, photochemical oxidation method, ultrasonic-ultraviolet combined method and other physical and chemical oxidation methods, membrane filtration method, and so on [15]. To sum up, the basic principles of the above pretreatment and advanced treatment methods are nothing more than four functions, such as adsorption, oxidation, biodegradation, and membrane filtration [16].

Persulfate (PS) advanced oxidation technology is a technology that utilizes PS to produce strong oxidizing hydroxyl radicals (·OH) under certain conditions to degrade organic pollutants in water [17]. This technology has the advantages of a fast reaction speed, low selectivity, the ability to treat difficult-to-degrade or non-biodegradable organic pollutants, and no secondary pollution, making it an effective water treatment technology [18]. However, PS advanced oxidation technology also has some limitations, such as the high cost of PS itself, fewer activation methods, and low stability in water [19].

**Figure 1:**
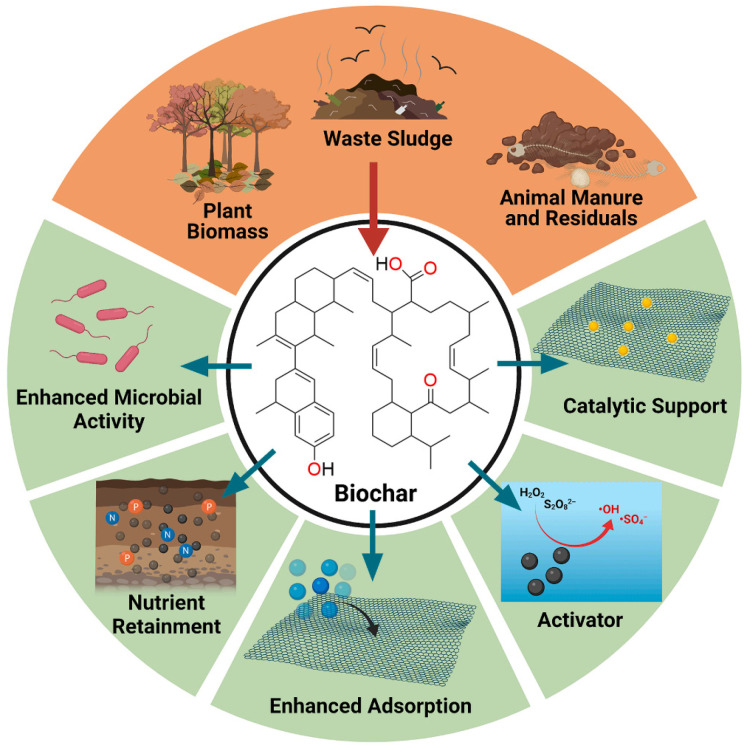
Summary of major sources of biochar feedstock and its applications [21]. Reproduced with permission from [Yuan J, Wen Y, Dionysiou D D, Sharma V K, Ma X.], Chemical Engineering Journal]; published by [ELSEVIER], [2022].

In order to overcome these limitations, in recent years, researchers put forward an innovative solution that entailed loading nZVI on BC to form a new persulfate catalyst, namely, BC-nZVI [20]. The typical sources of biochar feedstocks and its applications are summarized in Figure 1 [21]. This composite material has multiple advantages: (1) As a carrier, BC can provide a large amount of surface area and pore structure, increase the loading and dispersion of nZVI, and prevent its aggregation and oxidation [22]. (2) BC can adsorb organic pollutants in water, making it closer to nZVI and improving activation efficiency [23]. (3) BC can regulate the pH value of the solution, and promote the activation of PS and the generation of hydroxyl radicals [24]. (4) BC can capture some hydroxyl radicals, reduce their consumption of nZVI, and extend the lifespan of the catalyst [25]. This new catalyst, BC-nZVI, has advantages such as a low cost, simple preparation, good activation effect, and convenient regeneration and utilization, and it has broad application prospects.

Generally speaking, compared with other perspective articles, this paper analyzes the potential of BC-nZVI more comprehensively, including its excellent activation effect, cost-effectiveness, and sustainability. In addition, this paper also pays attention to the applicability of BC-nZVI in different environmental conditions and emphasizes its wide application prospect. Through an in-depth perspective of this field, we aim to provide valuable reference and inspiration for future research and promote the further development of this technology.

## 2. Preparation Method and Characterization Techniques of BC-nZVI

BC-nZVI is a novel environmental remediation material that utilizes biochar as a carrier to evenly disperse nZVI on its surface or within its pores, thereby enhancing the stability, activity, and reaction selectivity of nZVI. The preparation method and characterization techniques of BC-nZVI are crucial factors influencing its performance and applications, and this section provides a comprehensive review thereof.

### 2.1. Preparation Methods

BC-nZVI possesses advantages such as a high specific surface area, high reactivity, strong adsorption capacity, and low cost, making it effective for the removal of heavy metals, organic pollutants, and microorganisms from water and soil. This section reviews the principles, advantages, disadvantages, and influencing factors of various preparation methods for BC-nZVI, including the following.

#### 2.1.1. Physical Adsorption Method

The physical adsorption method is a simple preparation technique that utilizes the pore structure and surface functional groups of biochar to adsorb nZVI through electrostatic forces, van der Waals forces, or hydrogen bonds. This method is convenient to operate and does not require the use of reducing agents or other chemical reagents. However, the loading capacity and stability of nZVI are relatively low, making it prone to detachment or oxidation [26,27].

#### 2.1.2. Chemical Reduction Method

The chemical reduction method is a commonly used preparation technique that utilizes reducing agents (such as sodium borohydride, sodium sulfite, etc.) to reduce iron salts solution into nZVI, which is then deposited on the surface or within the pores of BC. This method can achieve a high loading capacity of nZVI and good dispersibility. However, it requires the use of toxic or flammable reducing agents, which may result in secondary pollution or safety hazards [28].

#### 2.1.3. Co-Precipitation Method

The co-precipitation method is an improved preparation technique that utilizes alkaline solutions (such as sodium hydroxide, ammonia, etc.) to precipitate Fe^2+^ and Fe^3+^ ions from iron salt solutions as Fe(OH)_2_ and Fe(OH)_3_, which are then mixed with BC. Under the protection of inert or reducing gases, Fe(OH)_2_ and Fe(OH)_3_ are thermally treated to reduce them to nZVI. This method avoids the use of organic reducing agents, reducing secondary pollution, while also enhancing the stability and reactivity of nZVI [29].

#### 2.1.4. Heat Treatment Method

The heat treatment method is a one-step preparation technique that involves impregnating iron salts onto biomass, followed by high-temperature pyrolysis under anaerobic conditions to simultaneously produce BC and nZVI. This method is simple to operate and cost-effective, but nZVI quality and dispersibility may be poor and influenced by biomass type and pyrolysis conditions [26].

#### 2.1.5. Other Methods

In addition to the aforementioned common preparation methods, there are also some other methods such as microwave-assisted, ultrasound-assisted, electrochemical, etc. These methods utilize external energy sources such as microwaves, ultrasound, or electric fields to promote the generation and loading of nZVI, enhancing its efficiency and effectiveness. However, they also require more instrumentation and energy consumption [30].

### 2.2. Characterization Techniques

BC-nZVI is a novel material with a high capability to degrade organic pollutants in water. To understand its structure, morphology, specific surface area, and the particle size of nano zero-valent iron, a series of characterization techniques are required. This section reviewed the characterization techniques for BC-nZVI, including XRD, SEM, TEM, and BET, discussing their principles, functions, and application scopes.

#### 2.2.1. X-ray Diffraction (XRD)

X-ray diffraction is a common material characterization technique that provides information on the crystalline structure of samples. By passing X-rays through the sample, a portion of the X-rays will be scattered at different angles, forming an X-ray diffraction pattern. The analysis of X-ray diffraction patterns can determine information such as the crystal structure, lattice constants, and crystal size of the sample. In the study of biochar-supported nano zero-valent iron, XRD can be used to determine the crystal structure and lattice constants of nano zero-valent iron, further understanding its properties. Additionally, XRD can be utilized to identify structural types and changes in the crystal structure of biochar-supported nano zero-valent iron [31].

#### 2.2.2. Scanning Electron Microscopy (SEM)

Scanning electron microscopy is a commonly used technique for surface morphology analysis. It scans the surface of the sample and detects the electrons reflected from the sample surface, forming images of the sample surface morphology [32]. SEM has advantages such as high resolution, a large depth of field, and a wide field of view, making it widely applicable in surface morphology analysis. In the study of biochar-supported nano zero-valent iron, SEM can be used to observe the morphology of the biochar surface and nano zero-valent iron, understanding the surface morphology and distribution of biochar-supported nano zero-valent iron. Additionally, SEM can be used to observe the size and dispersibility of nano zero-valent iron [31].

#### 2.2.3. Transmission Electron Microscopy (TEM)

Transmission electron microscopy is a high-resolution characterization technique for analyzing the internal structure and composition of materials. By passing an electron beam through the sample, information about the interaction between the electron beam and the sample is detected, forming images of the internal structure of the sample. In the study of biochar-supported nano zero-valent iron, TEM can be used to observe the internal structure of biochar and the distribution of nano zero-valent iron. TEM can provide information such as particle size, morphology, and the size distribution of nano zero-valent iron, and also observe the interaction and dispersibility between nano zero-valent iron and biochar [33].

#### 2.2.4. BET Surface Area Analysis

BET surface area analysis is a widely used technique for measuring the specific surface area of materials. By adsorbing specific gases (such as nitrogen) onto the sample surface and measuring the adsorption amount and pressure of the gas, the specific surface area of the material can be determined. BET surface area analysis is an important method for studying the adsorption performance and surface properties of adsorbent materials. In the study of biochar-supported nano zero-valent iron, BET can be used to measure the specific surface area of biochar and nano zero-valent iron. By measuring the specific surface area, the distribution of active sites, pore size distribution, and pore volume of biochar and nano zero-valent iron can be understood, further analyzing their application effects in the catalytic degradation of organic pollutants in water [34].

In summary, there are many characterization techniques for biochar-supported nano zero-valent iron, including XRD, SEM, TEM, BET, etc. Different techniques have different principles, functions, and application scopes, and a comprehensive approach involving multiple techniques is necessary for studying the structure and properties of biochar-supported nano zero-valent iron.

## 3. Mechanism and Influencing Factors of BC-nZVI Activation of PS for the Degradation of Organic Pollutants in Water

### 3.1. Mechanism of BC-nZVI-Activated PS to Degrade Organic Pollutants in Water

PS is a strong oxidant that can undergo reduction reactions on the surface of nZVI to generate sulfate radicals (SO_4_^−^·) and hydroxyl radicals (·OH). These radicals have a strong oxidation ability and can effectively degrade organic pollutants in water [35]. The active sites on the surface of nZVI (such as Fe^0^, Fe^2+^, Fe^3+^, etc.) participate in the reduction reaction of PS while also being corroded by PS, resulting in a decrease in the activity and stability of nZVI (Equations (1)–(5)) [36].

Fe^0^ → Fe^2+^ + 2e^−^ (1)

Fe^0^ + S_2_O_8_^2−^ → Fe^2+^ + 2SO_4_^2−^ (2)

Fe^0^ + H_2_O +0.5O_2_ → Fe^2+^ + 2OH^−^ (3)

Fe^0^ + 2H_2_O → Fe^2+^ + H_2_ + 2OH^−^ (4)

S_2_O_8_^2−^ + Fe^2+^ → Fe^3+^ + SO_4_^2−^ + SO_4_^−^ (5)

BC is a porous carbon material obtained from the low-temperature pyrolysis of biomass, characterized by a high specific surface area, abundant oxygen-containing functional groups, and persistent free radicals [21]. BC can effectively load nZVI to form nZVI /BC composite materials, improve the dispersion and stability of nZVI, and reduce the aggregation and oxidation of nZVI. The oxygen-containing functional groups and persistent free radicals on the surface of BC can form chemical bonds or physical adsorption with the surface of nZVI, preventing the corrosion effect of PS on nZVI and extending its service life [37]. Numerous micropores and mesopores on the surface of BC can provide adsorption sites for persulfate radicals, increase their lifespan in the reaction system, and improve their oxidation efficiency against organic pollutants [38]. At the same time, the oxygen-containing functional groups and persistent radicals on the surface of BC can also undergo electron transfer or radical transfer reactions with persulfate radicals, forming more active oxidants such as singlet oxygen (^1^O_2_) and superoxide radicals (O_2_^−^), further enhancing the oxidation ability of the reaction system [39]. The free radicals and nonradicals activated by reaction system can be expressed by Equations (6)–(10). Firstly, S_2_O_8_^2−^ was bound to biochar via chemical bonds and the electron transfer within the biochar–S_2_O_8_^2−^ complex resulted in the generation of SO_4_^−^· (Equations (6) and (7)). The formation of superoxide radicals could be attributed to the activation of PS by biochar (Equations (8)–(10)). The HO_2_^−^ radical that emerged during the PS activation would react with S_2_O_8_^2−^ to produce the O_2_^−^· radical (Equations (8) and (9)). HO_2_^−^ radical could also decompose and form the O_2_^−^· radical (Equation (10)). BC has a rich pore structure and polar surface functional groups, which can effectively adsorb organic pollutants in water [40].

BC + S_2_O_8_^2−^ → BC − S_2_O_8_^2−^ (6)

BC − S_2_O_8_^2−^ → BC + S_2_O_8_^2−^ (7)

S_2_O_8_^2−^ + 2H_2_O → HO_2_^−^ + 2SO_4_^2−^ + 3H^+^ (8)

S_2_O_8_^2−^ + HO_2_^−^ → SO_4_^−^· + SO_4_^2−^ + O_2_^−^· + H^+^ (9)

HO_2_· → O_2_^−^· + H^+^ (10)

The adsorption effect of BC on organic pollutants is mainly related to factors such as specific surface area, pore size, and the type and content of surface functional groups [41]. In the nZVI activation system loaded with BC, the adsorption effect of BC can concentrate organic pollutants in the reaction system, improve the contact opportunities between organic pollutants and nZVI and PS, and promote the degradation of organic pollutants. The surface functional groups and persistent free radicals of BC can interact with ions, molecules, and free radicals in the reaction medium, regulate physical and chemical parameters such as the pH value, ion strength, and dissolved oxygen content of the reaction medium, and affect the rate and efficiency of the activation reaction [42]. For example, the carbonate anhydride functional group of BC can undergo an acid–base neutralization reaction with H^+^ in the reaction medium, maintaining the stability and neutral environment of the reaction medium, which is conducive to the activation reaction.

### 3.2. The Main Reaction Pathway of BC-nZVI-Activated PS for the Degradation of Organic Pollutants in Water

The catalytic activity of BC-nZVI is mainly influenced by various factors, including surface oxygen-containing functional groups, defects, persistent free radicals, and metal atoms. Among these influencing factors are specific catalytic sites that can promote the catalytic process through free or non-free radical pathways, thereby achieving the efficient activation of refractory organic pollutants and producing highly active oxidation species, such as sulfate radicals (SO_4_^−^·), hydroxyl radicals (·OH), and superoxide radicals (O_2_^−^·). These oxidized species can undergo redox reactions with organic pollutants, resulting in the structural destruction and mineralization of the latter [43,44]. The main reaction pathways for the degradation of organic pollutants in water by BC-nZVI-activated PS include the following.

#### 3.2.1. Free Radical Pathway

**Figure 2:**
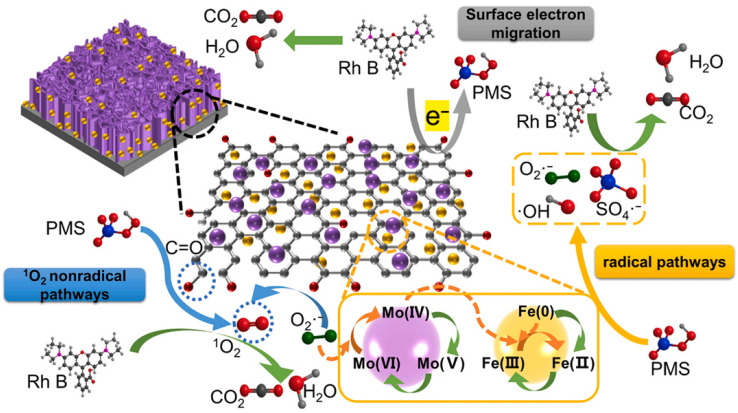
Mechanism of free radical pathway and non-free radical pathway in the nZVI/MoS2 BC+PS system [45]. Reproduced with permission from [Sun H, Zhang B, Wang N, Zhang N, Ma Y, Zang L, Li Z, Xue R], Journal of Environmental Chemical Engineering]; published by [ELSEVIER], [2023].

Metal atoms or persistent free radicals on the surface of BC-nZVI can directly undergo electron transfer reactions with PS, generating free radicals such as SO_4_^−^· and ·OH (Equations (1)–(4)). These free radicals have a strong oxidation ability and can quickly attack high electron density parts of organic pollutants, such as aromatic rings, double bonds, heteroatoms, etc., causing ring opening, bond breaking, or substitution reactions, as shown in Figure 2 [45].

#### 3.2.2. Non-Free Radical Pathway

Oxygenated functional groups or defects on the surface of BC-nZVI can form complexes or intermediates with PS and directly oxidize organic pollutants through single electron transfer or proton transfer reactions without generating free radicals [46]. For example, PMS showed high reactivity towards TCs in livestock wastewater without the generation of SO_4_^−^· [47]. Meanwhile, as for the singlet oxygen, it was reported that singlet oxygen could be generated by the recombination of O_2_^−^· radical and the reaction between O_2_^−^· and ·OH (Equations (11)–(18)). The SO_4_^−^· could also react with HO_2_^−^ radical to generate the ^1^O_2_ (Equation (19)). In addition, the C=O groups in biochar could interact with PS through the formation of a dioxirane intermediate to further give rise to the singlet oxygen. This approach has high selectivity and reaction stoichiometry efficiency and can effectively remove some organic pollutants that are not sensitive to free radicals, such as fluoroquinolone antibiotics.

SO_4_^−^· + OH^−^ → SO_4_^2−^ + ·OH (11)

·OH + ·OH → H_2_O_2_ (12)

·OH + H_2_O_2_ → HO_2_· + H_2_O (13)

O_2_^−^· + ·OH → ^1^O_2_ + OH^−^ (14)

2O_2_^−^· + 2OH^−^ → H_2_O_2_ + ^1^O_2_ (15)

2O_2_^−^· + ·2H_2_O → H_2_O_2_ + 2OH^−^ + ^1^O_2_ (16)

HO_2_· + O_2_^−^· → ^1^O_2_ + HO_2_^−^ (17)

·OH + O_2_^−^· → ^1^O_2_ + OH^−^ (18)

HO_2_· + SO_4_^−^· → HSO_4_^−^ + ^1^O_2_ (19)

### 3.3. Main Factors Affecting the Activation Performance of BC-nZVI

The main influencing factors on the activation performance of BC/nZVI include catalyst dosage, PS concentration, pH value, dissolved oxygen, anions, etc.

The catalyst dosage is an essential factor affecting the activation performance of BC/nZVI. Generally speaking, the pollutants’ removal rate increases as the catalyst dosage increases. However, when a specific critical value is reached, increasing the catalyst dosage does not significantly improve the removal rate of pollutants and may even have an inhibitory effect. Deng et al. [48] found that when BC/nZVI was used as a Fenton-like catalyst to remove sulfamethazine (SMT), when the dosage of nZVI/BC increased from 0.4 g/L to 1.2 g/L, the removal rate of SMT increased from 44.02% to 74.04%. However, when the dosage of nZVI/BC was further increased to 2 g/L, the removal efficiency of SMT did not improve, but decreased slightly instead. The concentration of PS is also a key factor affecting the activation performance of BC/nZVI. PS can provide strong oxidizing sulfate radicals and promote the oxidative degradation of pollutants. However, excessive PS concentration can lead to the consumption of free radicals and the passivation of the nZVI surface, thereby reducing the activation performance of BC/nZVI [49]. The pH value is another important factor affecting the activation performance of BC/nZVI. pH value affects not only the charge state and solubility of pollutants but also the surface charge and corrosion rate of nZVI. In general, a low pH value is beneficial for producing more electrons and hydrogen atoms on the surface of nZVI, thereby enhancing its reducing activity. However, a low pH value can also accelerate the corrosion and oxide film formation on the surface of nZVI, thereby reducing its activation performance. Dong et al. [50] found that weak acid conditions were favorable for the amination and dechlorination of para-Nitrochlorebenzene (P-NCB) in a study of the catalytic reduction of P-NCB by Fe-Pd bimetallic nanoparticles. Under these conditions, H_2_ produced by iron corrosion reaction was adsorbed on the surface of Pd and dissociated into H^+^, which accelerated the degradation process of p-NCB. However, when the pH value was 3.0, the reaction efficiency did not continuously improve as expected. Because the strong corrosion of iron led to the loss of catalyst, a lower pH value was not the most suitable condition for p-NCB reduction.

Dissolved oxygen has a dual effect on the activation performance of BC/nZVI. On the one hand, dissolved oxygen can promote the production of more free radicals and hydroperoxides on the surface of nZVI, thereby enhancing its oxidation activity. On the other hand, dissolved oxygen can also accelerate the corrosion and passivation of the nZVI surface, thereby reducing its activity [51]. Anions are another critical factor affecting the activation performance of BC/nZVI, and different types and concentrations of anions have different effects on the activation performance of BC/nZVI. Some anions, such as carbonate, sulfate, phosphate, etc., can form complexes or precipitates with the surface of nZVI, thereby inhibiting its activation performance. Other anions, such as chloride and bromine ions, can react or synergistically interact with the surface of nZVI, thereby enhancing its activation performance [43].

Different preparation methods and parameters can affect the morphology, structure, composition, and performance of BC-nZVI, thereby affecting its catalytic activity and stability. Generally, a higher temperature, longer time, a more significant proportion, and stronger reducing agents are beneficial for generating more nZVI particles, thereby improving their activation effect [52]. In addition, the concentration and dosing method of PS can affect the generation and distribution of hydroxyl radicals, thereby affecting degradation efficiency and selectivity. In addition, increasing the concentration of PS appropriately and adding PS in batches are beneficial for improving the degradation effect [53].

## 4. Application Study on BC-nZVI-Activated PS for the Degradation of Organic Pollutants in Water

**Figure 3:**
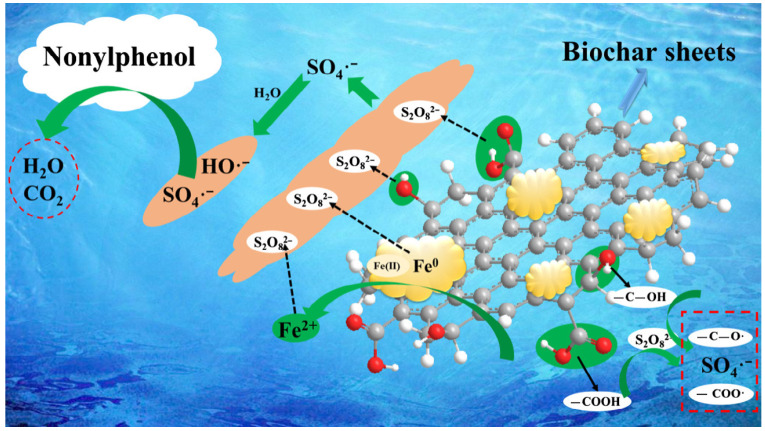
Proposed mechanism of NP degradation by the nZVI/BC nanocomposite activation of persulfate [35]. Reproduced with permission from [Hussain I, Li M, Zhang Y, Li Y, Huang S, Du X, Liu G, Hayat W, Anwar N], Chemical Engineering Journal]; published by [ELSEVIER], [2017].

Phenolic pollutants are a common type of organic pollutants in water, mainly from petrochemicals, pharmaceuticals, and agriculture industries. The sources of phenolic pollutants, their atmospheric sources produced simultaneously during biomass burning processes, and specific mechanisms can be found in the conversion of catechol to 4-nitrocatechol. Phenolic pollutants, including catechol, are emitted during biomass burning and combustion processes, contributing to the formation of 4-nitrocatechol in the atmosphere through reactions with NO_3_ and N_2_O_5_ [54,55]. Phenolic pollutants have characteristics such as strong toxicity, complex degradation, and carcinogenicity, causing severe harm to human health and the environment. BC-nZVI-activated PS can effectively degrade phenolic pollutants in water, such as phenol, cresol, chlorophenol, etc. During this process, BC can provide many surface-active sites, increase the loading and dispersion of nZVI, improve the activation efficiency and stability of PS, and adsorb organic matter in water, enhancing the degradation effect. Figure 3 illustrates the mechanism of phenolic pollutant (NP) degradation by the nZVI/BC nanocomposite activation of persulfate [56].

Wang et al. [57] investigated the use of green nano zero-valent iron (G-nZVI) supported on biochar for the removal of p-nitrophenol (PNP) from water. PNP is a toxic pollutant emitted from various industrial sources, and conventional removal methods often involve high energy consumption or lead to secondary pollution. G-nZVI, activated by potassium persulfate (PDS), is a low-cost, highly efficient, and environmentally friendly technology that has been extensively researched. However, the tendency of nZVI to aggregate affects its activity, thus necessitating its immobilization onto other materials to form new composite materials.

**Figure 4:**
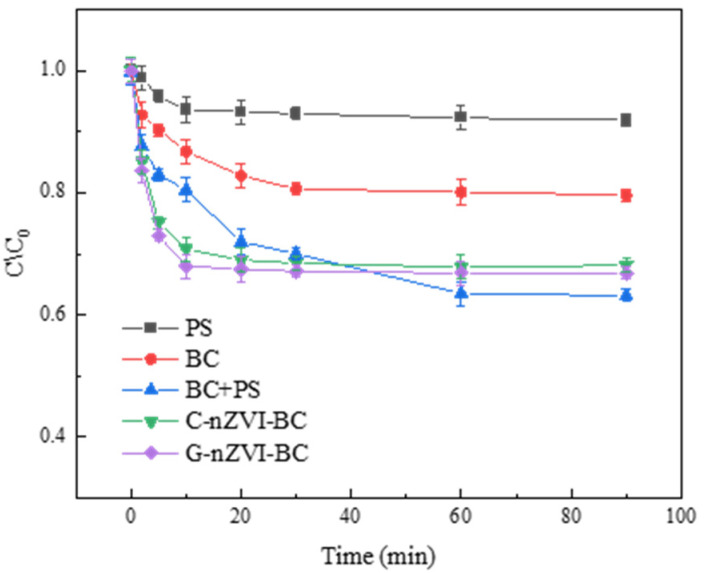
Degradation rate of PNP by different systems [57]. Reproduced with permission from [Wang B, Zhu C, Ai D, Fan Z], Journal of Hazardous Materials]; published by [ELSEVIER], [2021].

In this study, biochar was utilized as a carrier for G-nZVI, significantly enhancing the removal efficiency of PNP. The article examined the removal efficiency of PNP by five systems (PS, BC, C-nZVI-BC, G-nZVI-BC, and BC/PS), as shown in Figure 4 [57]. The results revealed that when PS or BC was used alone, the removal rates of PNP were very low, at 8.27% and 20.09%, respectively. In contrast, in systems incorporating nZVI, the removal rates of PNP were improved, with C-nZVI-BC and G-nZVI-BC reaching 31.85% and 32.68%, respectively. The BC/PS system exhibited the highest removal rate, at 36.71%, indicating a synergistic effect between biochar and potassium persulfate, where biochar could activate potassium persulfate to generate sulfate radicals due to the reaction between carboxyl and hydroxyl functional groups on the surface of biochar and potassium persulfate.

The study suggested that both free radical and non-free radical pathways play a role in the activation and degradation of PNP in the G-nZVI-BC/PS system, with the free radical pathway being the main degradation mechanism. This system efficiently removes PNP under pH conditions ranging from 3.06 to 9.23. Moreover, in the degradation of PNP in the G-nZVI-BC/PDS system, various transformation products were formed, including maleic acid, hydroquinone, catechol, p-Benzoquinone, and phenol, indicating the successful degradation of PNP into smaller molecules through processes such as the formation of polyhydroxy compounds, ring-opening reactions, and oxidation reactions leading to the generation of carbon dioxide and water.

The study also highlighted the good reusability and practical water treatment effects of G-nZVI-BC, demonstrating its broad application potential in water treatment. Applications of this research include the use of G-nZVI-BC as an effective and reusable catalyst for PNP degradation, indicating promising prospects in practical water treatment applications. The advantages of G-nZVI-BC in PNP removal demonstrate its effectiveness in treating water pollutants. Potential drawbacks may include certain cost and condition control requirements; however, overall, G-nZVI-BC exhibits high application potential.

Dye pollutants are a type of organic pollutant widely present in water, mainly from textiles, printing and dyeing, and papermaking industries. Dye pollutants have the characteristics of high chromaticity, difficult biodegradation, and mutagenicity, causing severe chromaticity pollution and ecological toxicity to water bodies. BC-nZVI-activated PS can effectively degrade dye pollutants in water, such as methylene blue, rhodamine B, methyl orange, etc. During this process, BC can increase the specific surface area and porosity of nZVI, improve the contact area and permeability of PS, and adsorb dye molecules in water, enhancing the degradation effect [58].

**Figure 5:**
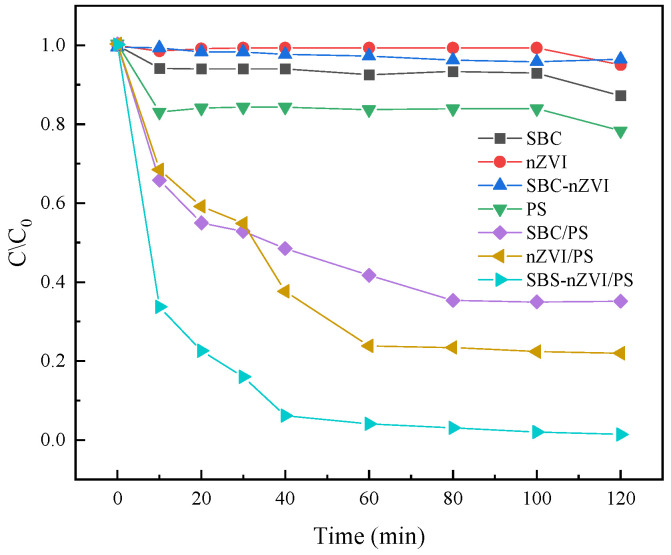
Degradation rate of AO II by different systems [59]. Reproduced with permission from [Liu H, Hu M, Zhang H, Wei J], Surfaces and Interfaces]; published by [ELSEVIER], [2022].

Liu et al. [59] used a biosynthetic method to couple straw biochar (SBC) with nZVI to form SBC-nZVI composite materials for application in wastewater treatment, specifically for removing dye contaminants, which exhibited the activation and degradation performance of model dye Acid Orange II (AO II). The experimental results are shown in Figure 5 [59]. The results showed that the SBC-nZVI/PS system was able to efficiently remove AO II in a short period, with a removal rate of 99.33%, significantly higher than the single SBC (63.94%) and nZVI (76.72%) systems. The SBC-nZVI composite material forms sulfate radicals SO_4_^−^· as the main species responsible for the degradation of AO II dye during the activation process. This indicates that SBC-nZVI composite materials have excellent activation degradation performance. According to the analysis, SBC can function as an electron carrier, enhancing electron transfer efficiency, while nZVI tends to aggregate due to its large specific surface area, thereby affecting its activation efficiency. The advantages of the SBC-nZVI composite material include an enhanced catalytic activity for persulfate, the improved dispersibility of nZVI, protection from oxidation, and increased electron transfer efficiency. Additionally, it exhibits good reusability, stability during storage, and broad applicability for various types of dyes. However, it is important to note that an excessive carbon to iron ratio may decrease catalytic activity, and high concentrations of persulfate can induce a quenching reaction. Furthermore, sulfate radicals are identified as the primary radical species responsible for contaminant removal. In summary, the SBC-nZVI composite material offers significant advantages for dye degradation in wastewater treatment due to its synergistic effects, stability, and efficient contaminant removal properties.

Pesticides are a common type of organic pollutant in water, mainly derived from the use and discharge of pesticides in agricultural production. Pesticide pollutants have the characteristics of strong toxicity, difficult degradation, and high residue, posing a potential threat to the aquatic ecological environment and human health. BC-nZVI-activated PS can effectively degrade pesticide pollutants such as acetochlor and flusilazole (or fluconazole) in water. BC can increase the loading and stability of nZVI, improve the activation efficiency and dispersibility of PS, and adsorb organic matter in water, enhancing the degradation effect [60].

**Figure 6:**
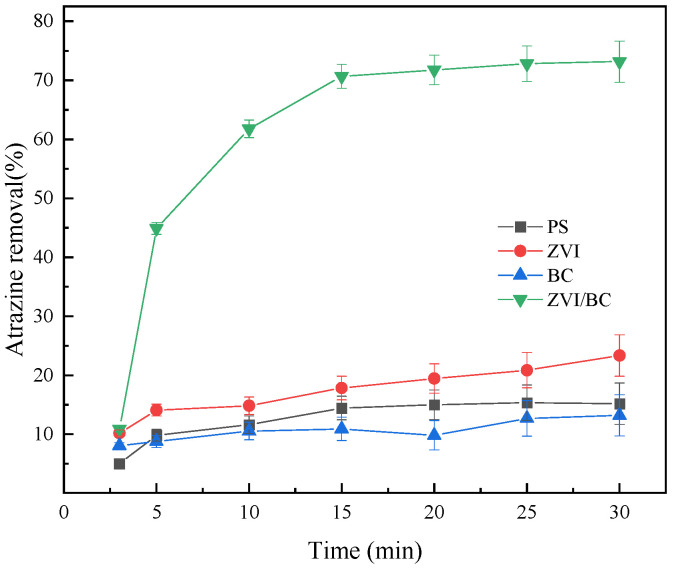
Degradation rate of atrazine by different systems [61]. Reproduced with permission from [Jiang Z, Li J, Jiang D, Gao Y, Chen Y, Wang W, Cao B, Tao Y, Wang L, Zhang Y], Environmental Research]; published by [ELSEVIER], [2020].

Jiang et al. [61] used zero-valent iron and biochar composite materials (nZVI/BC) as catalysts for PS to study its removal effect on the common herbicide atrazine in water. The application lies in efficiently degrading atrazine in water, offering an environmentally friendly solution for pollution remediation. The experimental results are shown in Figure 6 [61]. The experiment showed that the nZVI/BC-PS system can effectively degrade atrazine, with a degradation rate of 73.47% within 30 min and 83.77% under optimal conditions. Free radical capture experiments determined that SO_4_^−^· and ·OH were the main active oxidation species for atrazine degradation. The transformation products formed during the degradation of atrazine in the ZVI/BC-PS system include dealkylation, alkyl oxidation, and dechlorination–hydroxylation based on the proposed degradation pathways identified in the study. The researchers further analyzed the degradation pathways of atrazine in the ZVI/BC-PS system, including dealkylation, alkylation, and dechlorination–hydroxylation reactions. This study suggests that ZVI/BC, serving as a catalyst for persulfate (PS), presents an efficient and environmentally friendly technology for atrazine removal from water, which holds significant importance in safeguarding water quality and ecosystems, and reducing human health risks. Among its advantages, the composite system exhibits a high atrazine removal percentage within a short timeframe, demonstrating rapid and effective degradation capabilities. Through response surface methodology, optimized conditions are determined, thereby enhancing process efficiency. The active species generated during persulfate activation play a dominant role in atrazine degradation, underscoring the composite’s effectiveness in herbicide removal. As for potential drawbacks, the proper preparation and characterization of composite materials may be time-consuming, posing a logistical challenge. Additionally, further research is warranted to gain insights into the long-term stability and scalability of the ZVI/BC-activated persulfate system for atrazine removal.

In addition to phenolic, dye, and pesticide pollutants, there are other types of organic pollutants in water, such as antibiotics, benzene, aldehydes, ketones, etc. These organic pollutants have different properties and sources and require different treatment methods. BC-nZVI-activated PS can effectively degrade these organic pollutants and has broad application prospects [62].

**Figure 7:**
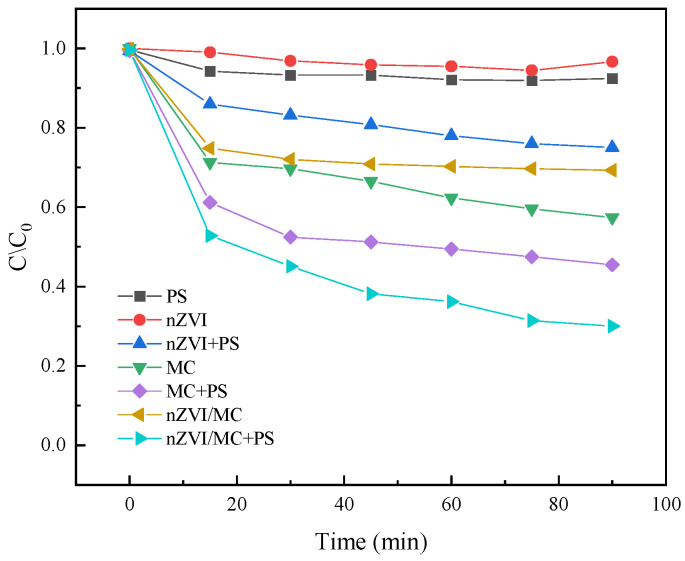
Degradation rate of SMZ by different systems [63]. Reproduced with permission from [Zhang L, Shen S], Journal of Industrial and Engineering Chemistry]; published by [ELSEVIER], [2020].

Zhang et al. [63] studied the process of nZVI/MC (mesoporous carbon) composite material activating PS to degrade sulfamethazine (SMZ). The application of this study lies in efficiently removing harmful organic compounds like SMZ from water bodies, thereby mitigating environmental and health risks associated with their presence. The removal efficiency of SMZ under different reaction conditions is shown in Figure 7 [63]. The results showed that the removal efficiency of SMZ using nZVI or PS alone was lower (3.2% and 7.4%, respectively) due to the small specific surface area of nZVI, the low redox potential of PS, and the lack of effective catalysts. When nZVI is combined with PS, the removal efficiency of SMZ is significantly improved (24.8%), indicating that nZVI can effectively activate PS to produce strong oxidizing free radicals. In addition, when MC and PS were combined, the removal efficiency of SMZ was 53.9%, which was attributed to the synergistic effect of MC on SMZ adsorption and PS activation. The adsorption and removal rates of SMZ by MC and nZVI/MC were 42.1% and 30.6%, respectively, indicating that the coverage of nZVI on the surface of MC reduced the specific surface area of MC. Finally, the nZVI/MC+PS process had the highest SMZ removal efficiency (69.5%), mainly due to the efficient activation of PS by the nZVI/MC catalyst, where nZVI participates in redox reactions and electron transfer processes. The degradation process of sulfamethazine (SMZ) generates several transformation products. Four intermediates were identified during the degradation process with mass-to-charge ratios (*m*/*z*) of 215, 245, 124, and 309. These intermediates corresponded to different degradation pathways of SMZ. The intermediate with *m*/*z* 215 (4-(2-imino-4,6-dimethylpyrimidin-1(2H)-yl) aniline, C_12_H_14_N_4_) was attributed to the Smiles-type rearrangement reaction. The aniline moiety can be attacked by sulfate radicals (SO_4_^−^·) and/or hydroxyl radicals (·OH), leading to the formation of an aniline radical cation. Additionally, the degradation product with *m*/*z* 124 (4,6-dimethylpyrimidin-2-amine, C_6_H_9_N_3_) was formed through different bond breakages, while the nitration product with *m*/*z* 309 (4-nitro-sulfamethazine, C_12_H_14_N_4_O_4_S) was a result of the oxidation of the aniline moiety under the attack of SO_4_^−^· and/or ·OH. These degradation pathways, including Smiles-type rearrangement and nitration, highlight the transformation products generated during the degradation of SMZ. The use of nZVI/MC as an activator of PS has shown promise in treating organic pollutants, highlighting its potential in water treatment technologies.

For an easy reading, the application of the BC-nZVI/PS system in the degradation of organic compounds in water is summarized briefly in Table 1.

## 5. Conclusions

BC-nZVI-activated PS technology is a new type of water treatment technology that uses BC as a carrier to disperse nZVI on its surface, forming highly active composite materials. By activating PS, strong oxidizing free radicals are generated, effectively degrading organic pollutants in water. Research has proved that BC-nZVI-activated PS can effectively degrade phenolic, dye, and pesticide pollutants in water, such as phenol, cresol, chlorophenol, methylene blue, rhodamine B, methyl orange, acetochlor, and flusilazole. The introduction of BC plays an essential role in catalyst composite materials, as it provides abundant surface-active sites, increases the loading and dispersion of nZVI, and enhances the adsorption of organic compounds in water, enhancing the degradation effect.

This perspective highlighted the differences in the degradation efficiency of organic pollutants by different catalyst composite systems. The synergistic effect between BC and PS can activate PS to generate sulfate free radicals, enhancing the degradation effect of phenolic organic pollutants. When dealing with dye pollutants, the BC-nZVI composite material exhibits excellent activation and degradation performance, efficiently removing methylene blue and other dyes by improving electron transfer efficiency and increasing specific surface area. In treating pesticide pollutants, BC-nZVI composite materials play an excellent activation role in the activation process of PS, achieving the efficient removal of pesticides such as acetochlor, which is of great significance for protecting water source quality and ecosystems, and reducing human health risks.

## 6. Future Directions

In summary, BC-nZVI-activated PS exhibits the potential for widespread application in water treatment. By optimizing the combination of catalyst composite materials and reaction conditions, degradation efficiency and stability can be further improved. Future research should focus on finding more economically feasible and environmentally friendly catalyst composite materials and conducting in-depth research on the degradation mechanisms of different organic pollutants.

For example, in terms of in-depth catalyst development, research work should focus on the synthesis of catalyst composites that are both economical and environmentally friendly. This involves screening various raw materials and optimizing the preparation process to improve the catalytic activity and stability. At the same time, in terms of comprehensive pollutant analysis, in order to effectively combat water pollution, it is necessary to study the degradation mechanism of different organic pollutants. This includes the use of advanced analytical techniques such as mass spectrometry and spectroscopy to identify reaction intermediates and degradation pathways. Meanwhile, on the issue of environmental impact assessment, in order to ensure the environmental protection of BC-nZVI-activated PS technology, researchers should conduct life cycle assessment and toxicity study. This will provide a comprehensive view of the ecological footprint and potential risks of this technology. In addition, from the perspective of expanding scale and practical application, it is very important to bridge the gap between laboratory scale and practical application. Research should focus on developing scalable technology and pilot-scale application to evaluate the feasibility and efficiency of this technology under realistic conditions. Lastly, from the point of interdisciplinary cooperative research, it is very important to encourage cooperation among scientists, engineers, and environmental experts. By bringing together different perspectives, we can develop comprehensive solutions, which can not only meet the pollution challenge, but also consider the broader environmental and social impacts. All this will help promote the practical application of BC-nZVI-activated PS technology in water treatment and provide new solutions for pollution control and the protection of water bodies.

## Figures and Tables

**Table 1:** Application of the BC-nZVI/PS system in the degradation of organic compounds in water.

Pollutant Type	Degradation Effectiveness	Degradation Products	Advantages and Disadvantages	Practical Application Direction	Data Sources
Phenolic pollutants	1. G-nZVI-BC/PS effectively removes PNP from water. 2. Alone, PS or BC has low removal rates for PNP (8.27% and 20.09%, respectively). 3. Adding nZVI significantly boosts PNP removal (C-nZVI-BC: 31.85%; G-nZVI-BC: 32.68%). 4. BC/PS shows the highest removal rate (36.71%), indicating synergy.	In G-nZVI-BC/PDS, PNP degradation produces maleic acid, hydroquinone, catechol, p-Benzoquinone, and phenol.	Advantages: G-nZVI-BC is reusable and effective in water treatment. Disadvantages: Potential cost and condition control requirements.	G-nZVI-BC is a promising catalyst for PNP degradation in water treatment, showcasing its potential in tackling water pollutants.	[57]
Dye pollutants	SBC-nZVI/PS efficiently removes AO II dye from wastewater, achieving a remarkable 99.33% removal rate in a short time.	Sulfate radicals (SO_4_^−^·) are the primary species responsible for AO II dye degradation in the SBC-nZVI composite material.	Advantages: SBC-nZVI exhibits enhanced catalytic activity, improved dispersibility, protection from oxidation, and good reusability. It also applies broadly to various dye types. Disadvantages: Excessive carbon to iron ratio may reduce catalytic activity, and high persulfate concentrations can lead to quenching reactions.	SBC-nZVI holds promise for dye degradation in wastewater treatment due to its synergistic effects, stability, and efficient contaminant removal.	[59]
Pesticide pollutants	nZVI/BC-PS effectively degrades atrazine, achieving 73.47% degradation within 30 minutes and 83.77% under optimized conditions.	Transformation products during atrazine degradation include dealkylation, alkyl oxidation, and dechlorination–hydroxylation.	Advantages: High atrazine removal in a short time, enhanced by optimized conditions. Active species from persulfate activation play a key role. Disadvantages: Time-consuming preparation and characterization of composite materials. Further research needed for long-term stability and scalability.	ZVI/BC as a PS catalyst offers efficient and eco-friendly atrazine removal, vital for safeguarding water quality and ecosystems, and reducing health risks.	[61]
Other organic pollutants	1. The combination of nZVI and PS improves SMZ removal to 24.8%, compared to 3.2% with nZVI alone and 7.4% with PS alone. 2. Adding mesoporous carbon (MC) enhances removal to 53.9% due to synergistic effects. 3. nZVI/MC+PS achieves highest removal at 69.5%, facilitating redox reactions and electron transfer processes.	SMZ degradation generates transformation products with various mass-to-charge ratios, indicating different degradation pathways.	Advantages: nZVI/MC effectively activates PS, enhancing organic pollutant removal. It offers synergistic effects and shows potential in water treatment. Disadvantages: nZVI coverage on MC may reduce specific surface area, affecting adsorption rates. Proper preparation and characterization require time and resources.	nZVI/MC shows promise in efficiently treating organic pollutants like SMZ, highlighting its potential in water treatment technologies and mitigating environmental and health risks.	[63]

## Data Availability

Not applicable.

## References

[1] Madhav S., Ahamad A., Singh A.K., Kushawaha J., Chauhan J.S., Sharma S., Singh P. (2020). Water pollutants: Sources and impact on the environment and human health. Sensors in Water Pollutants Monitoring: Role of Material.

[2] Morin-Crini N., Lichtfouse E., Liu G., Balaram V., Ribeiro A.R.L., Lu Z., Stock F., Carmona E., Teixeira M.R., Picos-Corrales L.A. (2022). Worldwide cases of water pollution by emerging contaminants: A review. Environ. Chem. Lett..

[3] Mushtaq N., Singh D.V., Bhat R.A., Dervash M.A., Hameed O.B. (2020). Freshwater contamination: Sources and hazards to aquatic biota. Freshwater Contamination: Sources and Hazards to Aquatic Biota.

[4] Huang W., Song B., Liang J., Niu Q., Zeng G., Shen M., Deng J., Luo Y., Wen X., Zhang Y. (2021). Microplastics and associated contaminants in the aquatic environment: A review on their ecotoxicological effects, trophic transfer, and potential impacts to human health. J. Hazard. Mater..

[5] Singh J., Yadav P., Pal A.K., Mishra V. (2020). Water pollutants: Origin and status. Sensors in Water Pollutants Monitoring: Role of Material.

[6] Saravanan A., Kumar P.S., Jeevanantham S., Karishma S., Tajsabreen B., Yaashikaa P., Reshma B. (2021). Effective water/wastewater treatment methodologies for toxic pollutants removal: Processes and applications towards sustainable development. Chemosphere.

[7] Inobeme A., Adetunji C.O., Mathew J.T., Okonkwo S., Bamigboye M.O., Ajai A.I., Afoso E., Inobeme J.A. (2023). Advanced nanotechnology for the degradation of persistent organic pollutants. Microbial Degradation and Detoxification of Pollutants Berlin.

[8] Savoca D., Pace A. (2021). Bioaccumulation, biodistribution, toxicology and biomonitoring of organofluorine compounds in aquatic organisms. Int. J. Mol. Sci..

[9] Akhtar N., Syakir Ishak M.I., Bhawani S.A., Umar K. (2021). Various natural and anthropogenic factors responsible for water quality degradation: A review. Water.

[10] Pandian A.M.K., Rajamehala M., Singh M.V.P., Sarojini G., Rajamohan N. (2022). Potential risks and approaches to reduce the toxicity of disinfection by-product–A review. Sci. Total Environ..

[11] Gopinath K.P., Rajagopal M., Krishnan A., Sreerama S.K. (2021). A review on recent trends in nanomaterials and nanocomposites for environmental applications. Curr. Anal. Chem..

[12] Yaqoob A.A., Parveen T., Umar K., Mohamad Ibrahim M.N. (2020). Role of nanomaterials in the treatment of wastewater: A review. Water.

[13] Sun S., Jiang T., Lin Y., Song J., Zheng Y., An D. (2020). Characteristics of organic pollutants in source water and purification evaluations in drinking water treatment plants. Sci. Total Environ..

[14] Zhao H.-X., Zhang S., Zhang T.-Y., Zhu Y.-P., Pan R.-J., Xu M.-Y., Zheng Z.-X., Hu C.-Y., Tang Y.-L., Xu B. (2024). Comparison of four pre-oxidants coupled powdered activated carbon adsorption for odor compounds and algae removal: Kinetics, process optimization, and formation of disinfection byproducts. Sci. Total Environ..

[15] Nabavi E., Sabour M., Dezvareh G.A. (2022). Ozone treatment and adsorption with granular activated carbon for the removal of organic compounds from agricultural soil leachates. J. Clean. Prod..

[16] Ojha S., Tripathi S.M., Vishwakarma P.K., Mishra S. (2024). Pharmaceuticals in the Water: Emerging Concerns and Innovative Remediation Solutions. Curr. Green Chem..

[17] Raza M.A., Farwa U., Mumtaz M.W., Mukhtar H., Rashid U. (2024). Homogeneous Advanced Oxidation Processes. Advanced Oxidation Processes for Micropollutant Remediation.

[18] Hussain I., Zhang Y., Huang S., Du X. (2012). Degradation of p-chloroaniline by persulfate activated with zero-valent iron. Chem. Eng. J..

[19] Liu Y., Liu L., Wang Y. (2021). A Critical review on removal of gaseous pollutants using sulfate radical-based advanced oxidation technologies. Environ. Sci. Technol..

[20] Huang P., Zhang P., Wang C., Du X., Jia H., Sun H. (2023). P-doped biochar regulates nZVI nanocracks formation for superefficient persulfate activation. J. Hazard. Mater..

[21] Yuan J., Wen Y., Dionysiou D.D., Sharma V.K., Ma X. (2022). Biochar as a novel carbon-negative electron source and mediator: Electron exchange capacity (EEC) and environmentally persistent free radicals (EPFRs): A review. Chem. Eng. J..

[22] Li S., Yang F., Li J., Cheng K. (2020). Porous biochar-nanoscale zero-valent iron composites: Synthesis, characterization and application for lead ion removal. Sci. Total Environ..

[23] Fan C., Chen N., Qin J., Yang Y., Feng C., Li M., Gao Y. (2020). Biochar stabilized nano zero-valent iron and its removal performance and mechanism of pentavalent vanadium (V (V)). Colloids Surf. A Physicochem. Eng. Asp..

[24] Kemmou L., Frontistis Z., Vakros J., Manariotis I.D., Mantzavinos D. (2018). Degradation of antibiotic sulfamethoxazole by biochar-activated persulfate: Factors affecting the activation and degradation processes. Catal. Today.

[25] Lai C., Huang F., Zeng G., Huang D., Qin L., Cheng M., Zhang C., Li B., Yi H., Liu S. (2019). Fabrication of novel magnetic MnFe_2_O_4_/bio-char composite and heterogeneous photo-Fenton degradation of tetracycline in near neutral pH. Chemosphere.

[26] Awang N.A., Wan Salleh W.N., Aziz F., Yusof N., Ismail A.F. (2023). A review on preparation, surface enhancement and adsorption mechanism of biochar-supported nano zero-valent iron adsorbent for hazardous heavy metals. J. Chem. Technol. Biotechnol..

[27] Ken D.S., Sinha A. (2020). Recent developments in surface modification of nano zero-valent iron (nZVI): Remediation, toxicity and environmental impacts. Environ. Nanotechnol. Monit. Manag..

[28] Li Q., Zhao S., Wang Y. (2021). Mechanism of oxytetracycline removal by coconut shell biochar loaded with nano-zero-valent iron. Int. J. Environ. Res. Public. Health.

[29] Xu D., Fan X., Chen Q., Qiao S., Zhang J., Yang Y., Wang H., Zhang L., Hou J. (2022). Removal of nitrogen and phosphorus from water by sludge-based biochar modified by montmorillonite coupled with nano zero-valent iron. Water Sci. Technol..

[30] Li L., Hu J., Shi X., Fan M., Luo J., Wei X. (2016). Nanoscale zero-valent metals: A review of synthesis, characterization, and applications to environmental remediation. Environ. Sci. Pollut. R..

[31] Liu C., Lu J., Tan Y., Chen B., Yang P. (2022). Removal of U (VI) from wastewater by sulfhydryl-functionalized biomass carbon supported nano-zero-valent iron through synergistic effect of adsorption and reduction. Mater. Sci. Eng. B.

[32] Vos K., Vandenberghe N., Elsen J. (2014). Surface textural analysis of quartz grains by scanning electron microscopy (SEM): From sample preparation to environmental interpretation. Earth-Sci. Rev..

[33] Aragaw T.A., Bogale F.M., Aragaw B.A. (2021). Iron-based nanoparticles in wastewater treatment: A review on synthesis methods, applications, and removal mechanisms. J. Saudi Chem. Soc..

[34] Shaheen S.M., Mosa A., Abdelrahman H., Niazi N.K., Antoniadis V., Shahid M., Song H., Kwon E.E., Rinklebe J. (2022). Removal of toxic elements from aqueous environments using nano zero-valent iron-and iron oxide-modified biochar: A review. Biochar.

[35] Hussain I., Li M., Zhang Y., Li Y., Huang S., Du X., Liu G., Hayat W., Anwar N. (2017). Insights into the mechanism of persulfate activation with nZVI/BC nanocomposite for the degradation of nonylphenol. Chem. Eng. J..

[36] Chen Z., Cao W., Bai H., Zhang R., Liu Y., Li Y., Song J., Liu J., Ren G. (2023). Review on the degradation of chlorinated hydrocarbons by persulfate activated with zero-valent iron-based materials. Water Sci. Technol..

[37] Liang W., Wang G., Peng C., Tan J., Wan J., Sun P., Li Q., Ji X., Zhang Q., Wu Y. (2022). Recent advances of carbon-based nano zero valent iron for heavy metals remediation in soil and water: A critical review. J. Hazard. Mater..

[38] Ye S., Xiong W., Liang J., Yang H., Wu H., Zhou C., Du L., Guo J., Wang W., Xiang L. (2021). Refined regulation and nitrogen doping of biochar derived from ramie fiber by deep eutectic solvents (DESs) for catalytic persulfate activation toward non-radical organics degradation and disinfection. J. Colloid. Interface Sci..

[39] Pi Z., Li X., Wang D., Xu Q., Tao Z., Huang X., Yao F., Wu Y., He L., Yang Q. (2019). Persulfate activation by oxidation biochar supported magnetite particles for tetracycline removal: Performance and degradation pathway. J. Clean. Prod..

[40] Liang L., Xi F., Tan W., Meng X., Hu B., Wang X. (2021). Review of organic and inorganic pollutants removal by biochar and biochar-based composites. Biochar.

[41] Qiu B., Shao Q., Shi J., Yang C., Chu H. (2022). Application of biochar for the adsorption of organic pollutants from wastewater: Modification strategies, mechanisms and challenges. Sep. Purif. Technol..

[42] Ji J., Yuan X., Zhao Y., Jiang L., Wang H. (2022). Mechanistic insights of removing pollutant in adsorption and advanced oxidation processes by sludge biochar. J. Hazard. Mater..

[43] Zhao C., Shao B., Yan M., Liu Z., Liang Q., He Q., Wu T., Liu Y., Pan Y., Huang J. (2021). Activation of peroxymonosulfate by biochar-based catalysts and applications in the degradation of organic contaminants: A review. Chem. Eng. J..

[44] Shi Q., Deng S., Zheng Y., Du Y., Li L., Yang S., Zhang G., Du L., Wang G., Cheng M. (2022). The application of transition metal-modified biochar in sulfate radical based advanced oxidation processes. Environ. Res..

[45] Sun H., Zhang B., Wang N., Zhang N., Ma Y., Zang L., Li Z., Xue R. (2023). Refractory organics removal in PMS and H_2_O_2_/PMS oxidation system activated by biochar/nZVI/MoS_2_ composite: Synthesis, performance, mechanism and dosing methods. J. Environ. Chem. Eng..

[46] Zhao G., Zou J., Chen X., Liu L., Wang Y., Zhou S., Long X., Yu J., Jiao F. (2021). Iron-based catalysts for persulfate-based advanced oxidation process: Microstructure, property and tailoring. Chem. Eng. J..

[47] Chen J., Xu J., Liu T., Qian Y., Zhou X., Xiao S., Zhang Y. (2020). Selective oxidation of tetracyclines by peroxymonosulfate in livestock wastewater: Kinetics and non-radical mechanism. J. Hazard. Mater..

[48] Deng J., Dong H., Zhang C., Jiang Z., Cheng Y., Hou K., Zhang L., Fan C. (2018). Nanoscale zero-valent iron/biochar composite as an activator for Fenton-like removal of sulfamethazine. Sep. Purif. Technol..

[49] Zhang Y., Jiang Q., Jiang S., Li H., Zhang R., Qu J., Zhang S., Han W. (2021). One-step synthesis of biochar supported nZVI composites for highly efficient activating persulfate to oxidatively degrade atrazine. Chem. Eng. J..

[50] Zhu F., Wu Y., Liang Y., Li H., Liang W. (2020). Degradation mechanism of norfloxacin in water using persulfate activated by BC@ nZVI/Ni. Chem. Eng. J..

[51] Karim S., Bae S., Greenwood D., Hanna K., Singhal N. (2017). Degradation of 17α-ethinylestradiol by nano zero valent iron under different pH and dissolved oxygen levels. Water Res..

[52] Zhou H., Ma M., Zhao Y., Baig S.A., Hu S., Ye M., Wang J. (2022). Integrated green complexing agent and biochar modified nano zero-valent iron for hexavalent chromium removal: A characterisation and performance study. Sci. Total Environ..

[53] Xi M., Cui K., Cui M., Ding Y., Guo Z., Chen Y., Li C., Li X. (2021). Enhanced norfloxacin degradation by iron and nitrogen co-doped biochar: Revealing the radical and nonradical co-dominant mechanism of persulfate activation. Chem. Eng. J..

[54] Rana M.S., Bradley S.T., Guzman M.I. (2023). Conversion of Catechol to 4-Nitrocatechol in Aqueous Microdroplets Exposed to O_3_ and NO_2_. ACS EST Air.

[55] Rana M.S., Guzman M.I. (2022). Oxidation of Catechols at the Air–Water Interface by Nitrate Radicals. Environ. Sci. Technol..

[56] Hanafi M.F., Sapawe N. (2020). A review on the water problem associate with organic pollutants derived from phenol, methyl orange, and remazol brilliant blue dyes. Mater. Today: Proc..

[57] Wang B., Zhu C., Ai D., Fan Z. (2021). Activation of persulfate by green nano-zero-valent iron-loaded biochar for the removal of p-nitrophenol: Performance, mechanism and variables effects. J. Hazard. Mater..

[58] Pan X., Gu Z., Chen W., Li Q. (2021). Preparation of biochar and biochar composites and their application in a Fenton-like process for wastewater decontamination: A review. Sci. Total Environ..

[59] Liu H., Hu M., Zhang H., Wei J. (2022). Biosynthesis of stalk Biochar-nZVI and its catalytic reactivity in degradation of dyes by persulfate. Surf. Interfaces.

[60] Morillo E., Villaverde J. (2017). Advanced technologies for the remediation of pesticide-contaminated soils. Sci. Total Environ..

[61] Jiang Z., Li J., Jiang D., Gao Y., Chen Y., Wang W., Cao B., Tao Y., Wang L., Zhang Y. (2020). Removal of atrazine by biochar-supported zero-valent iron catalyzed persulfate oxidation: Reactivity, radical production and transformation pathway. Environ. Res..

[62] Kumar A., Singh E., Mishra R., Kumar S. (2022). Biochar as environmental armour and its diverse role towards protecting soil, water and air. Sci. Total Environ..

[63] Zhang L., Shen S. (2020). Adsorption and catalytic degradation of sulfamethazine by mesoporous carbon loaded nano zero valent iron. J. Ind. Eng. Chem..

